# In Vitro Wound Healing Potential of Stem Extract of* Alternanthera sessilis*

**DOI:** 10.1155/2018/3142073

**Published:** 2018-02-19

**Authors:** Katyakyini Muniandy, Sivapragasam Gothai, Woan Sean Tan, S. Suresh Kumar, Norhaizan Mohd Esa, Govindasamy Chandramohan, Khalid S. Al-Numair, Palanisamy Arulselvan

**Affiliations:** ^1^Laboratory of Vaccines and Immunotherapeutics, Institute of Bioscience, Universiti Putra Malaysia, Serdang 43400, Selangor, Malaysia; ^2^Department of Medical Microbiology and Parasitology, Faculty of Medicine and Health Sciences, Universiti Putra Malaysia, Serdang 43400, Selangor, Malaysia; ^3^Department of Nutrition and Dietetics, Faculty of Medicine and Health Sciences, Universiti Putra Malaysia, Serdang 43400, Selangor, Malaysia; ^4^Department of Community Health Sciences, College of Applied Medical Sciences, King Saud University, P.O. Box 10219, Riyadh 11433, Saudi Arabia; ^5^Muthayammal Centre for Advanced Research, Muthayammal College of Arts and Science, Rasipuram, Namakkal, Tamilnadu 637408, India

## Abstract

Impaired wound healing is one of the serious problems among the diabetic patients. Currently, available treatments are limited due to side effects and cost effectiveness. In line with that, we attempted to use a natural source to study its potential towards the wound healing process. Therefore,* Alternanthera sessilis* (*A. sessilis*), an edible and medicinal plant, was chosen as the target sample for the study. During this investigation, the wound closure properties using stem extract of* A. sessilis* were analyzed. Accordingly, we analyzed the extract on free radical scavenging capacity and the cell migration of two most prominent cell types on the skin, human dermal fibroblast (NHDF), keratinocytes (HaCaT), and diabetic human dermal fibroblast (HDF-D) to mimic the wound healing in diabetic patients. The bioactive compounds were identified using gas chromatography-mass spectrometry (GC-MS). We discovered that the analysis exhibited a remarkable antioxidant, proliferative, and migratory rate in NHDF, HaCaT, and HDF-D in dose-dependent manner, which supports wound healing process, due to the presence of wound healing associated phytocompounds such as Hexadecanoic acid. This study suggested that the stem extract of* A. sessilis* might be a potential therapeutic agent for skin wound healing, supporting its traditional medicinal uses.

## 1. Introduction

The chronic wound has become one of the most frightening phenomena in the world. Diabetic patients are at the highest risk to develop chronic wound due to the conditions such as high glucose level, neuropathy, poor blood circulation, and prolonged inflammation around the limbs that causes the healing to be delayed compared to normal patients [1]. The seriousness of the issue can be estimated with the tremendous increase in the number of diabetic patients annually, and the World Health Organization (2016) reported that 422 million adults were living with diabetes in 2014, compared to 108 million in 1980 which showed the terrifying rise from 4.7% to 8.5% in the adult population [2]. Another study conducted by the World Health Organization (2011) mentioned that this metabolic disease is estimated to rise from 171 million to 366 million in the year 2030 [3], which means it is estimated to be doubled in 15 years of time. Unfortunately, a recent global statistic showed that the number of diabetic patients in 2014, has surpassed the predicted number of diabetic patient in the year 2030, which demonstrates the tremendous increase in diabetic population worldwide. When the study is scaled down to Malaysia, there are predicted about 3.5 million of the population having this metabolic disease [4].

The skin is the largest organ, where, on an average adult, it accounts for about 16% of total body weight [5]. The ultimate function of the skin is to guard our body against external assaults by forming a protective barrier covering the body; therefore any injury or breakage on the skin should be amended quickly to provide continuous protection to our bodily system. Skin is made of two main layers, the outer layer and inner layer which are also known as epidermis and dermis, respectively. In vitro assessment of both epidermis and dermis layer of human skin has been commonly carried out using HaCaT cells, a human-derived immortalised keratinocytes cell line, and human dermal skin fibroblasts (HDFs) cell. The multilayered epidermis is composed of keratinocytes at its deepest layer, which will be constantly renewed by proliferating, differentiating, and migrating upward in the epidermis to replace the worn out cells due to abrasion. In a healthy skin condition, the rate of keratinocytes proliferation is equal to the rate of cell loss to the environment [6]. On the other hand, dermis is an underneath layer of the skin consisting of fibroblast that is essential in maintaining the structural integrity by secreting collagen in the extracellular matrix [7].

The damage happened on the surface of epithelial barrier and its underlying connective tissue, and it is described as the occurrence of wound. Wound healing is a natural phenomenon that involves complex processes to attain the skin's ordinary anatomical structure and functions. An ideal healing process varies in its duration as it implicates four sequentially overlap phases: (1) the coagulation and formation of platelet scab to cover the wound opening to prevent further blood loss or pathogen entry, (2) the rush of inflammatory cells to the wound site for protection and to fight against pathogens as well as to activate the skin cells, (3) the skin cells proliferate rapidly to replace the lost cells, and (4) the repairing of skin structure. These processes can be presented in single word units known as “homeostasis,” “inflammation,” “proliferation,” and “remodeling,” respectively [8]. In some healing processes, the wound fails to progress in a predictable, timely, and spatially coordinated manner and thus healing is delayed. This occurrence is mainly because of inflammation that is triggered and prolonged in an unusual duration due to stimuli, that is, physical (e.g., tissue injury), chemical (e.g., detergent), or biological (e.g., pathogen infection), thereby lengthening the inflammatory phase that eventually interrupts the normal recovery process [9]. The prevalence is likely to grow exponentially due to the elevation of the risky populations such as the elderly and the obese and the diabetic subjects.

In certain severe cases of nonhealing wound condition, amputation is essential to remove the necrotic tissue to prevent any further damage that may cause serious sickness or even death, but this removal of a body part is testified to affect the quality of the patients' lives as well as their emotion [36]. In the current vascular surgery practice, diabetes and its associated complication such as the chronic wound remain as the prime reason for people to necessitate amputation. However, this unwanted condition can be prevented if the wound can be healed; therefore, wound healing has become an important topic of current research. In the present time, there are many solutions available: (1) antibiotics to remove the microorganism, (2) hyperbaric oxygen therapy to supply the skin tissue with sufficient oxygen, (3) maggot therapy using* Lucilia (Phaenicia) sericata* [37] for detrimental purpose, and (4) topical dressings using creams such as silver sulfadiazine, bacitracin-zinc ointment, Santyl, Accuzyme, and fusidic acid [38, 39] as the therapeutics for healing process. Unfortunately, each of them has their own drawbacks such as the development of resistance towards the microorganism, the high cost of treatment, the unwillingness and disgust, and the development of allergic reaction, respectively. The development of allergic reaction from tropical dressings may vary from one individual to another due to the difference in their sensitivity reactions and health backgrounds [40]. Therefore, it is an urge to find an effective and safe alternative method of therapy, particularly from edible plants, which is believed to be minimally toxic [41]. In ancient time, folk healers used conventional methods to overcome delayed wound healing problems by using plants' preparations, as plants produce secondary metabolites such as phenols and flavonoid, saponin, and alkaloids. Secondary metabolites may not be useful for the plant's growth or its reproduction, but they give selective advantages to the organisms, believed and proven scientifically to have natural therapeutic properties [42]. In line with this demand, we conducted a study based on an edible plant,* A. sessilis*, which has been used in many parts of the world.

*A. sessilis* is locally known as the “sessile joyweed” and is a succulent herb, with branches of about 50 cm height from the root, and is prominently in wet places [43]. It is commonly eaten as a vegetable and taken in soups in many Asian countries particularly in India. Besides, it has also been used by the folk healers to cure headaches, heal burns, wash eyes, and deal with snakebites. In addition, it is also prepared into decoction to be taken orally, for febrifuge, as anticough, and to treat gastrointestinal complications such as diarrhea [44].* A. sessilis* is believed to be a promising therapeutic candidate for wound healing, as previous studies have described its various pharmacological activities such as antibacterial [45], antioxidant [46], and antiallergic [47] properties that are vital in a good healing process. Furthermore, anti-inflammatory associated bioactive constituents such as epigallocatechin, catechin, chlorogenic acid, 4-hydroxybenzoic, apigenin, vanillic acid, ferulic acid, ethyl gallate, and daidzein were reported in this plant; thus, this may justify the usage of this vegetable for this field of study [48]. Also, there is still a lack on reported studies about the wound healing potential of this plant's stem extract tested on normal and diabetic skin cell. Hence, this investigation could bridge the therapeutic gap that is essential for diabetic wound healing.

In this study, we intended to evaluate the effect of 90% hydroethanolic extract of stem part of* A. sessilis* towards the wound closure rate on (1) the normal human dermal fibroblast, (2) the normal keratinocytes, and (3) the diabetic human dermal fibroblast, along with hyphenated technique, gas chromatography-mass spectrometry (GC-MS), for the identification of organic biochemical components present in the stem extract of* A. sessilis*.

## 2. Methods

### 2.1. Plant Collection and Extract Preparation

The* A. sessilis *plant was purchased from a market at Serdang (Google Maps coordinate: 3°1′0.636′′N 101°42′46.069′′E) and it was authenticated by botanist, Dr. Shamsul Khamis, and deposited in the IBS Herbarium unit for further reference with the voucher specimen number (SK 2938/15). The stem part was isolated, cleaned, washed, dried, and grinded to form a fine powder. The powder of stem part of* A. sessilis* was macerated into 90% hydroethanolic solvent for 3 days and filtered. The maceration and the filtration steps were performed at room temperature. The filtrate collected was then evaporated to remove the solvent, through the rotary evaporator. The thickened filtrate was freeze dried and stored at −20°C until further use. Before each analysis being conducted, ethanolic crude extract of stem part was freshly prepared by dissolving in PBS premixed with 1% DMSO (dimethyl sulfoxide) and was further diluted into different concentrations with growth medium (DMEM) to cater for different assays.

### 2.2. Antioxidant Assay

The antioxidant or the free radical scavenging activity of the plant extract was measured by using 1,1-diphenyl-2-picrylhydrazyl (DPPH) assay. The stock of DPPH solution (0.1 mM in methanol) was prepared, and 150 *μ*L of DPPH solution was mixed with 150 *μ*L of sample or the standard (gallic acid) in methanol at preferable concentrations. The reaction mixture was mixed thoroughly and left in dark environment at room temperature for about 30 min. The reading was taken with the absorbance value of 517 nm. The percentage of inhibition of scavenging was determined by the following equation:

(1) $$\begin{array}{c} Radical\;\;scavenging\;\;activity\left(\%\right)=\frac{A0-A1}{A0}\times 100, \end{array}$$

where* A*0 is the absorbance of DPPH radicals with methanol, while the* A*1 is the absorbance of DPPH radicals with sample extract or standard.

The IC_50_ values of the extract and standard were determined. Experiments were performed in triplicate, and the data were presented in the average value.

### 2.3. Cell Culture Maintenance

NHDF, HDF-D, and HaCaT cells were acquired from American Type Culture Collection (ATCC, Manassas, VA, USA, PCS-201-012); ZenBio, Inc.; and Cell Lines Service, Germany, 300493, respectively. The cell lines were thawed and well maintained. Cells were cultured and maintained in high glucose Dulbecco's Modified Eagle Medium (DMEM) premixed with 10% fetal bovine serum and antibiotics (streptomycin 100 *μ*g/mL and penicillin 100 U/Ml) in a humidified 5% CO_2_ incubator at 37°C.

### 2.4. 3-(4,5-Dimethylthiazol-2-yl)-2,5-diphenyl Tetrazolium Bromide [MTT] Assays

NHDF, HaCaT, and HDF-D cells were seeded into 96-well plate at a density of 1 × 10^4^ (in 100 *μ*L of DMEM medium) per well and grown for 24 hrs. The medium was replaced with different concentrations of stem crude extract of 15.62, 31.25, 62.5, 125, 250, and 500 *μ*g/ml, and the plates were incubated for 24 h. 10 *μ*L of 5 mg/mL MTT reagent was then added to each of the wells and incubated for another 4 h. The purple formazan formed was solubilized by adding 100 *μ*L dimethyl sulfoxide to all the wells including control (without any treatment) and then swirled gently to mix well and was then kept in the dark place at room temperature for about 30 min. Microplate reader was used to read absorbance at 570 nm. The percentage cell viability (CV) was calculated using the formula below:

(2) $$\begin{array}{c} CV\left(\%\right)=\frac{Absorbance\;\;of\;\;test\;\;sample}{Absorbance\;\;of\;\;control}\times 100. \end{array}$$

Accordingly, graph of percentage of cell viability against concentrations was plotted. Experiments were performed in triplicate and the data were presented as mean ± SD (*n* = 3).

### 2.5. In Vitro Wound Scratch Assay

The migration rates of NHDF, HDF-D, and HaCaT cells were assessed by the scratch assay method. The cell density of “2 × 10^5^ cells” was seeded into each well of a 24-well plate and incubated with complete medium at 37°C and 5% CO_2_. After 24 h of incubation, the monolayer confluent cells were scrapped horizontally with a sterile P200 pipette tips. The debris was removed by washing with PBS. The cells were treated with ethanolic extract of* A. sessilis *stem with various concentration (12.5 *μ*g/mL, 25 *μ*g/mL, and 50 *μ*g/mL) by diluting with serum-free DMEM. The cells without treatment and treatment with allantoin (Sigma Aldrich, Germany) (50 *μ*g/ml) were used as the control and positive control, respectively. The scratch induced that represented wound was photographed at 0 h using phase contrast microscopy at ×40 magnification at 0 h, before incubation with the ethanolic extract of* A. sessilis* stem. After 24 hrs of incubation, the second set of images was photographed. To determine the migration rate, the images were analyzed using “imageJ” software, and percentage of the closed area was measured and compared with the value obtained at 0 h. An increase in the percentage of the closed area indicated the migration of cells. Experiments were performed in the triplicate manner and the data were recorded and analyzed statistically using SPSS.

(3) $$\begin{array}{c} Wound\;\;closure\left(\%\right)=\frac{\left(Measurement\;\;at\;\;0\;h-Measurement\;\;at\;\;24\;h\right)}{Measurement\;\;at\;\;0\;h}\times 100. \end{array}$$

### 2.6. Gas Chromatography-Mass Spectrometry

GC-MS analysis of the ethanolic extract of* A. sessilis *was performed using a QP-2010 Ultra GC-MS spectrometer (Shimadzu, Kyoto, Japan) fused with a BPx5 column (30 × 0.25 *μ*m ID × 0.25 *μ*m df). The oven temperature was programmed from 50°C at 0 min and increased to 300°C and remained constant for 10 min. The stem extract was diluted in methanol. Helium gas (99.999%) was used as a carrier gas with the following conditions: total flow: 11.8 mL/min, column flow: 0.8 mL/min, linear velocity: 32.4 cm/s, purge flow: 3.0 mL/min, and split ratio: 10. Mass spectra were taken with ion source temperature and interface temperature of 200°C and 250°C, respectively. The mass scan parameters had a start time of 2.5 min, with end time 93.0 min. The acquisition (ACQ) parameters involved the following conditions: scan event time: 0.10 s, scan speed: 10000, and mass range: 40 *m*/*z* to 700 *m*/*z*. The relative percentage of each compound was calculated by comparing its average peak area to the total amount of areas.

### 2.7. Statistical Analysis

The result was expressed as a mean ± SD, and the significance of results was analyzed using one-way ANOVA (ANOVA) SPSS version 21.0 software (SPSS, USA). Results obtained were compared with control and treated groups using Student's *t*-test. Differences between the groups were considered as statistically significant at ^*∗*^*P* < 0.05, ^*∗∗*^*P* < 0.01, and ^*∗∗∗*^*P* < 0.001 versus control group.

## 3. Results

### 3.1. Effect of Stem of* A. sessilis* on Free Radical Scavenging Action

**Figure 1:**
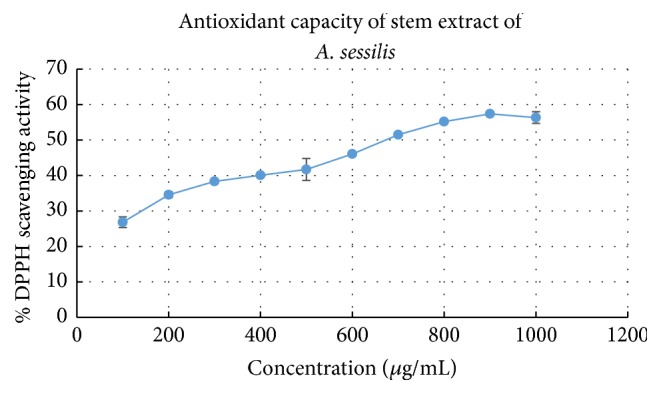
The DPPH scavenging action of stem extract of* A. sessilis* increased in a concentration-dependent manner. Values are mean ± SD (*n* = 3).

In the DPPH radical scavenging assay, the antioxidants presence reacted with the purple DPPH and converted into yellow coloured a,a-diphenyl-*β*-picryl hydrazine and the rate of conversion indicated the antioxidant potential of the sample. In the test, the scavenging activity increased in a concentration-dependent manner due to the scavenging capacity of the sample and was comparable to gallic acid. The IC_50_ value denotes the concentration required to scavenge 50% of the initial DPPH radicals. The IC_50_ value of the stem extract of* A. sessilis* was around 782 *μ*g/ml ± 29.9 *μ*g/mL; in comparison, the standard (gallic acid) had an IC_50_ of 31.34 ± 3.6 *μ*g/mL. According to the IC_50_ value, it shows that the scavenging action of the pure compound, gallic acid, is 25x more compared to the crude extract tested (Figure 1).

### 3.2. Effect of* A. sessilis* Stem Extract on the Cell Viability

**Figure 2:**
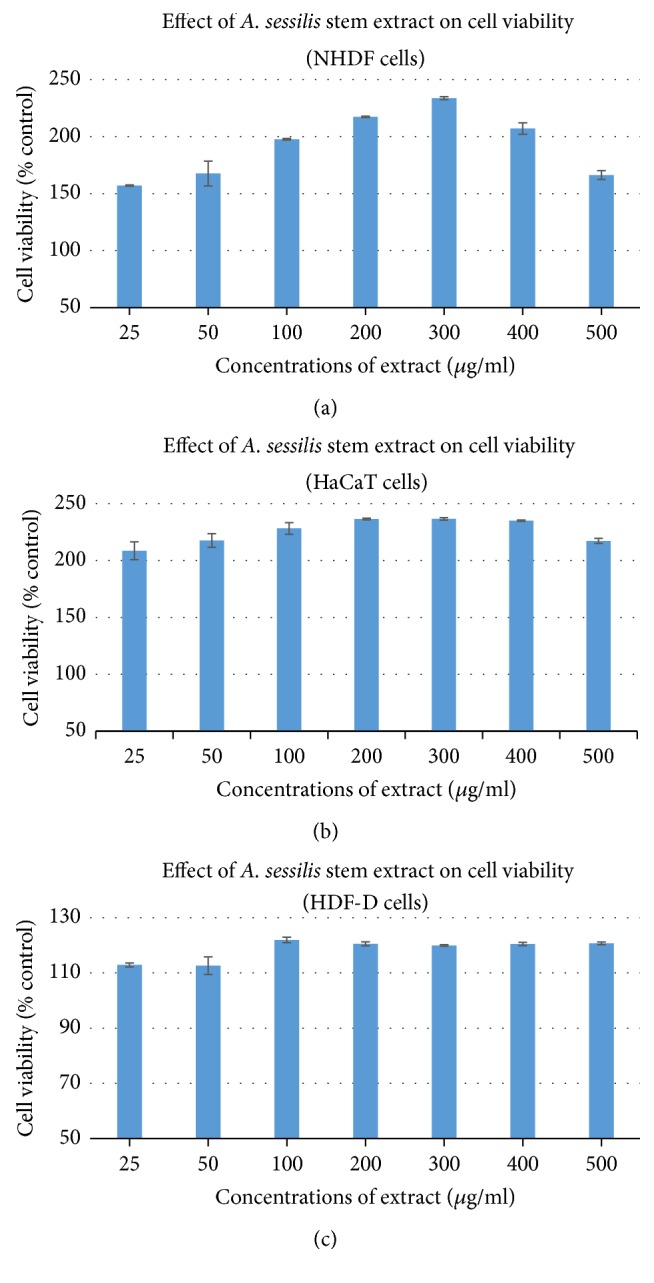
Effect of ethanolic extract of* A. sessilis* stem on viability of NHDF (a), HaCaT (b), and HDF-D (c). Cell viability following incubation with indicated concentrations of crude extract for 24 h was determined using the MTT assay. Cell viability is expressed as a percentage of untreated cells. Results shown in the graphs are mean ± SD obtained from triplicate experiments.

To select the optimal drug concentrations for the cell culture experiments, we investigated the effect of* A. sessilis *stem extract on cell viability after incubation for 24 hrs by 3-(4,5-dimethylthiazol-2-yl)-2,5-diphenyl tetrazolium bromide (MTT) reduction assay. As seen in Figures 2(a), 2(b), and 2(c), exposure of all the three types of cells to the concentrations up to 500 *μ*g/ml did not show noticeable toxicity as the cell viability remained above 80%. Even though pattern does not show a continuous increase in cell viability as the concentration increases, a slight decrease in cell viability was observed as the concentration increases; however it is still under the safe dosage range to be used for further biological experiments. It is noticeable that Figures 2(a) and 2(b), which present the normal cells, showed clearly more cell viability rate compared to the cell of Figure 2(c) which is the diabetic isolated cell.

### 3.3. Effect of* A. sessilis* Stem Extract on the Migration of Keratinocytes and Fibroblast Cells

Migration of cells play vital role in wound repair; thus scratch assay was conducted to observe the healing process to the test sample and the minimal active concentrations were chosen from the cell viability experiments. After 24 h exposure to the test sample, it was observed that the cell migrates towards the provisional gap induced. The values given were calculated based on the scratch coverage rate in the 24 hours. The images were shown in Figure 4 of NHDF (a), HaCaT (b), and HDF-D (c). Allantoin, a plant derived commercialized drug, was used as the positive control drug with the concentration of 50*μ*g/ml.

The control groups indicate the natural rate of migration of cell, without the influence of the ethanolic extract of* A. sessilis* stem or the standardized drug. The natural migration was noted to be the lowest in all the cell lines tested. The migration analysis shows that, for the NHDF (a), the treated cells with the lowest concentration of extract showed a significant increase in migration with the value of 59%, when compared with the control group which is only 32%. When the highest treatment group is compared, it showed a significant increase in migration with the migration rate of 86% when compared to control group, yet it is still slower with 3% difference when compared with positive control group.

**Figure 3:**
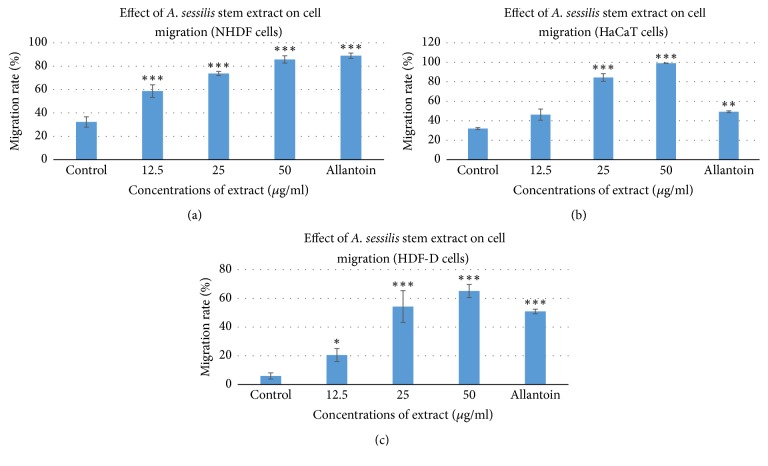
The migration rate in percentage for NHDF (a), HaCaT (b), and HDF-D (c) after treatment with stem extract of* A. sessilis *for 24 hrs. Quantitative analysis of the migration rate was analyzed with the use of ImageJ software. Data are expressed as mean ± standard deviation from three individual experiments. ^*∗*^*P* < 0.05, ^*∗∗*^*P* < 0.01, and ^*∗∗∗*^*P* < 0.001 versus control group.

On the other hand, for the HaCaT (b), the entire treated group even with the lowest concentration showed some significant increase in migration rate compared to control group. The highest concentration (50*μ*g/ml) of treatment of extract showed 99% in migration rate, and when it was compared with control and positive control group, it showed a clear increase with the rate of 209% and 156%, respectively, which is 2- and 1 1/2-fold (Figure 3).

In addition, HDF-D (c) showed the treatment for HDF isolated from diabetic source. The control group showed a very low migration, which is only 6%. It is the major problem faced in the wound healing field, among the diabetic patients. When allantoin is given, it increases the migration rate up to 51%. Our extract, with the same concentration, 50 *μ*g/ml, provided us with a strong positive result which is 65%, with the difference of 14% of migration rate between them. However, treatment of the extract for all the above cell lines showed increase in migration rate in a dose-dependent manner.

### 3.4. Identification of Bioactive Compounds through Output from GC-MS Analysis

The results pertaining to GC-MS analysis of the hydroethanolic extract of* A. sessilis *showed the presence of 50 prominent peaks in the chromatogram (Figure 5). Interpretation of the mass spectra and chromatograms was proposed with the equipment databases such as the National Institute Standard and Technique (NIST11s.lib) database and the Flavors and Fragrances of Natural and Synthetic Compounds (FFNSC1.3.lib). 20 out of 50 compounds, which correspond to 58.81% of the entire GC-MS chromatogram, were successfully identified. The retention time, the percentage of concentration of peak area, and the reported biological activities of the 20 compounds were presented in Table 1.

Of the twenty compounds identified, the most prevailing compounds were 2,4-dihydroxy-2,5-dimethyl-3(2H)-furan-3-one (8.92%), hexadecanoic acid <n-> (7.21%), 2-1,2,4-trioxolane,3-phenyl- (5.99%), palmitate <ethyl-> (5.65%), and L-glutamic acid (5.04%). Out of the 20 phytocompounds identified, 5 of them exhibited antioxidant capacity and 11 of them possessed anti-inflammatory potential while antimicrobial activities were determined in 10 of the compounds.

## 4. Discussion

Skin is the largest of the body's organs which ultimately forms the wall-like barrier separating our internal body from the outer environment. It protects us from dampness, cold, and sunlight, as well as notorious agent be it physical (pressure), chemical (detergent), or biological (excess proinflammatory cytokines, necrosis condition, hypoxic environment, and presence of pathogenic bacteria) that can be detrimental if it enters our body [49]. However, the level of protection is unlikely to be adequate if there is any breakage in the skin surface, which is also known as the wound. Therefore, occurrence of wound should be diagnosed, treated, and healed as soon as possible with proper medications. In this study, we investigated the effects of stem part of* A. sessilis* on wound healing with the expectation that this extract could induce skin regeneration.

Wound healing is characterized by the process of reepithelization that is associated with angiogenesis which means the process of blood vessel formation to supply the tissues with sufficient nutrients essential for proliferation, growth, and migration, in order to replace the devitalized and missing cellular structures and tissue layers [50]. Fibroblasts and keratinocytes, the main component of granulation tissue and epithelial barrier, respectively, are the dominant cells in wound closure mechanism [51]. As soon as homeostasis takes place, the fibroblast at the injury starts to secrete extracellular proteins that help the underlying keratinocytes to proliferate rapidly and after few days they migrate to the existing matrix replacing the fibrin scab formed into the collagen-rich matrix [52, 53]. Fibroblast's movement towards the provisional matrix is influenced mainly by the orientation as well as the composition of the matrix, and this condition is known as the “contact guidance” [54, 55]. Dermis is the layer of skin which is rich in blood supply and nutrients, while the epidermis is lacking them; thus their nourishment is provided from the dermis layer through the deepest layer of epidermis which is the stratum basale where keratinocytes proliferate being pushed up towards the skin surface [56]. The cells on the skin surface are constantly worn out and sloughed off in a process known as desquamation and they will be continuously replaced by the cells from the underlying layer of epidermis.

Several evidences showed that keratinocytes and fibroblast have excellent interactions in wound healing. One of them is that keratinocytes fuel the fibroblast to make growth factors, and this will then stimulate keratinocytes multiplications in a double paracrine manner [52, 57, 58]. Growth factors are the proteins such as Keratinocyte Growth Factor (KGF), Platelet Derived Growth Factor (PDGF), Vascular Endothelial Growth Factor (VEGF), Transforming Growth Factor Beta (TGF-*β*), and Granulocyte-Macrophage Colony-Stimulating Factor (GM-CSF) which play roles, by promoting angiogenesis, vascularization, and proliferation for nourishment for new cell growth to support wound healing processes. This showed the role of both the cells is equally important in wound healing process.

In line with that, we have studied this phenomenon by treating our plant extract on keratinocytes, dermal fibroblast, and diabetic dermal fibroblast to analyze its potential on diabetic wound healing. Furthermore, variant vulnerability of fibroblast and keratinocytes has been reported for other active compounds due to difference in its receptor signaling sensitivity [59]; therefore it is a promising approach for wound healing if we could identify an extract that promotes migration on both the cell types simultaneously.

Wound closure is a dynamic mechanism that involves a combination of proliferation and migration of skin cells at the wound site. Cytotoxicity or MTT assay applied in the study functions in multiple manner whereby the cell viability percentage denotes the suitable usage of concentrations for the upcoming treatments for assay as well as to measure the cell proliferation rate as an alternative to tritiated thymidine incorporation into DNA [60]. However, this cytotoxicity assay is specially made for cell metabolic measurement which quantifies the rate of reduction of tetrazolium dye MTT 3-(4,5-dimethylthiazol-2-yl)-2,5-diphenyltetrazolium bromide into formazan, by an enzyme that is only functional in actively respiring or, in other words, living cell. Nevertheless, this is applicable in the measurement of cell proliferation as the rate of living cells is calculated by comparison with the untreated group cell. On the other hand, besides proliferation study, for an in vitro investigation for wound closure analysis, scratch assay is a suitable yet inexpensive method to validate cell migration activity, and this analysis is greatly associated with the third phase of wound healing process which is proliferation that it mimics to some extent migration of cells during wound closure [61].

**Figure 4:**
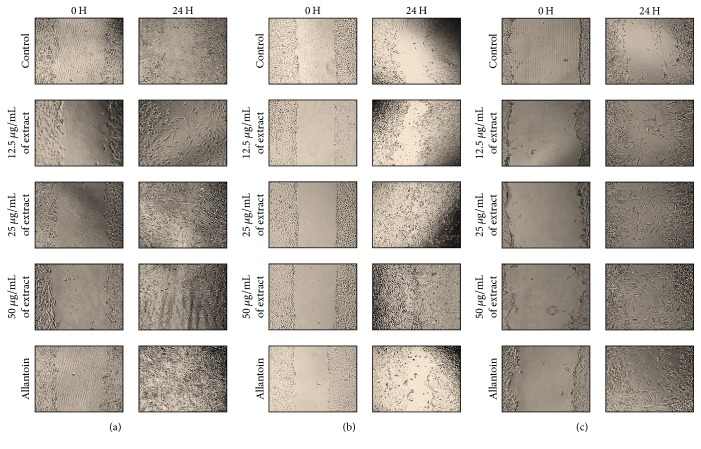
In vitro scratch assay (×40 magnification). NHDF (a), HaCaT (b), and HDF-D (c) cells were scratched and treated with and without treatment of varying concentrations of plant extract. Ethanolic extract of stem part of* A. sessilis* showed positive cell proliferation and cell migration as compared with control group (without treatment).

**Figure 5:**
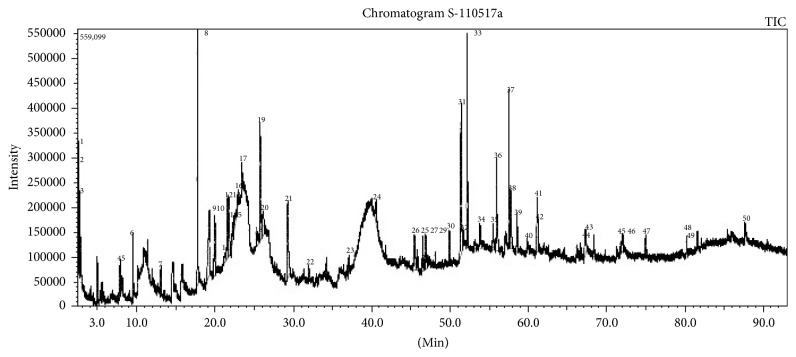
Gas chromatogram for stem extract of* A. sessilis*.

Figure 2 shows that the rate of cell viability is more than 100% and thus corroborates its functions in proliferation while Figure 3 proves that the extract could act on broad signaling receptors to promote proliferation and migration on HaCat as well as HDFs. Healing process is delayed among diabetic patients due to interrupted angiogenesis process; thus nourishment and growth factors synthesis is disrupted and results in lower rate of healing. This is supported with the data from Figures 4 and 3(c) that shows that current standardized medication is efficient for all the cell types, yet the migration rate is lower in HDF-D when compared with the NHDF. However, the* A. sessilis* stem extract induces almost equal rate of cell migration for NHDF and HDF-D. Conversely, when the rate of migration is compared between the NHDF and Hacat (Figures 4 and 3(b)), the same concentration seems to induce migration more on the NHDF and this might be the fact from previous study that mentioned that higher concentration is needed for stimulation of epithelial barrier while lower dosage is sufficient to fuel up the cellular compounds of connective tissue [62].

In a normal biological context, every living cell that undergoes aerobic metabolism produces reactive oxygen species (ROS) as its byproduct. ROS is essentially important for normal physiological process such as cell signaling but, in certain circumstances such as stresses, the level of ROS increases dramatically which is ruinous for the cell also for the organism overall in a larger scale. Generation of high amount of oxidative stress happens when the reactive oxygen species is not balanced up with its antioxidant capacity. Plant is an immobile living organism that is unable to escape from stresses such as drought and salinity, which builds up toxic materials in it, yet, to an extent, it is able to overcome the stresses and continue its sustainability. The reason is that they are equipped with highly efficient scavenging mechanism known as an antioxidative system, regulated by antioxidative compounds. The same is done by the skin cells, the production of free radicals as the byproducts of metabolism should be balanced up for the healthy cell maintenance critical for it to play its healing role [63].

In this present study, we used an extract of stem from* A. sessilis* and it is believed to be a minimally toxic source, as the plant is widely used as a vegetable at many regions of the globe throughout the world. According to previous reports,* A. sessilis* leaves have been investigated for its wound healing activity and it has demonstrated a positive result by stimulating epithelization on rat model [64]. Therefore, by using the same source of plant species, another uncommon part of the plant, which is the stem, was analyzed to fill an existing gap to confirm the overall plant's biological properties towards wound healing activity. A highly polar solvent ethanol was used to extract the plant's secondary metabolites by targeting phenolic compounds from the extract as it is one of the prominent groups of secondary metabolites of them. However, a very minimal volume of water was used to make up the solvent, as water supports the activity of enzyme polyphenol oxidase that degrades polyphenols [65]. In order to conserve the phenolic compounds that are believed to be active compounds for our study, we used alcohol as it does not affects the enzyme's activity. Methanol can be used as it is more polar than ethanol but its cytotoxic nature is unsuitable to be used and, therefore, ethanol was used instead of methanol and, at the same time, the slight increase in hydrophobicity assisted in the cell degradation and the solvent's penetration through the membrane of the cells, to extract the intracellular contents out [66].

Elevated glucose level among the diabetic patients causes physiological dysfunctions which results in the delay of healing process than normal individual without diabetes. Major factors that affect the healing ability are easy colonization of bacteria and generation of high amount of oxidative stress that prolongs the inflammatory phase. Consequently, the wound healing process remains in the inflammatory phase in an abnormally extended duration, without progressing to the next healing phase (proliferative phase) and subsequent phase thus delaying the healing process [67]. Due to the fact that prolonged inflammatory phase is one of the chief problems in healing process, an efficient therapeutic candidate should be rich in anti-inflammatory properties. Identified phytocompounds (Table 1) in the extract were reported to suppress anti-inflammatory pathways which are NF-*κ*B and MAPK [25, 28, 32]; therefore healing process can be proceeded to the next phases towards recovery.

It is reported that* Bacillus* sp.,* Staphylococcus aureus*, Methicillin-resistant* Staphylococcus aureus*,* Pseudomonas aeruginosa*,* Klebsiella pneumonia*,* Escherichia coli*,* Candida* sp., and* Aspergillus* sp. are the commonly isolated microorganisms from diabetic foot ulcer [68, 69]. One of the previous studies demonstrated antimicrobial effect of fatty acids on the mentioned microorganisms and hexadecanoic acid (palmitic acid) was believed to inhibit the growth of the all above stated pathogenic microorganisms [26]. Hence, antimicrobial potential of the extract can be predictable due to presence of few antimicrobial associated compounds. Not only that but also antioxidant properties clearly revealed the ability of radical scavenging activity as shown in Figure 1 and the presence of antioxidant related compounds in the extracts strengthens the justification that the extract possesses antioxidant activity.

## 5. Conclusion

**Figure 6:**
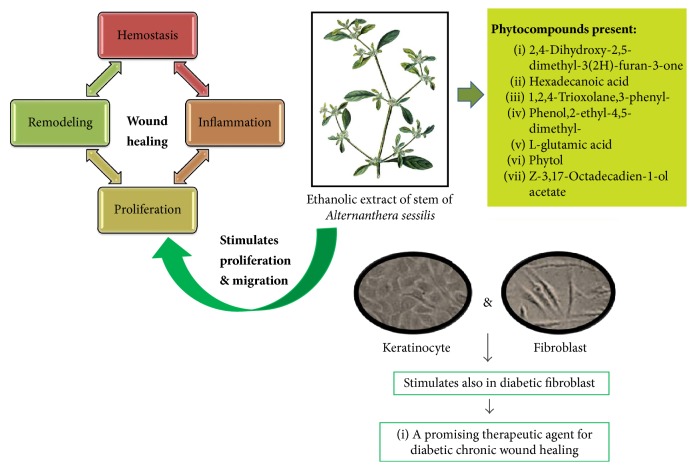


This report clearly demonstrated that ethanolic extract of stem part of* A. sessilis* was effective in enriching wound closure progression in normal and diabetic fibroblast cells, as well as keratinocytes. Thus, it may be potential natural products based candidate to elucidate the delayed wound healing issue among the diabetic patients. The bioactive compounds present in this crude extract have been successfully identified and confirmed by GC-MS and the corresponding biological activities of those compounds have supported the wound healing potential of* A. sessilis* stem extract. This extract shall be furthered for fractionation for detailed compound identification through various bioactive compound isolation techniques for the development as alternative natural therapeutic wound healing agent (Figure 6).

## Figures and Tables

**Table 1:** Total compounds identified in the crude extract of stem part of *A. sessilis* using GC-MS analysis with its retention time, peak area, and reported biological activities.

Peak number	Compounds	^*∗*^R.T	^*∗*^P.A (%)	Reported biological activities	Citations
1.	Pentanal <n->	2.612	1.95	*Antimicrobial *	[10].

2.	Formic acid, 2-propenyl ester	2.660	1.71	No reported activity	

4.	Butyrolactone	7.868	0.69	*Anti-inflammatory*	[11].

6.	Furfural <5-methyl->	9.532	1.3	*Antibacterial* *Antifungal*	[12, 13].

7.	Phenylacetaldehyde	13.139	1.09	*Antimicrobial* *Anti-inflammatory *Antineoplastic	[14, 15].

8.	2,4-Dihydroxy-2,5-dimethyl-3(2H)-furan-3-one	17.793	8.92	*Antioxidant*	[16].

9.	1,2,4-Trioxolane,3-phenyl-	19.295	5.99	*Antibacteria* *Antifungal* *Antiprotozoal* Antitumor *Immunomodulator*	[17].

19.	Phenol, 2-ethyl-4,5-dimethyl-	25.763	5.00	*Antibacterial Anti-inflammatory*	[18].

21.	L-glutamic acid	29.262	5.04	Acts as amino acid Fuel for metabolism Anticancer *Anti-inflammation Immunomodulator* Neurotransmitter	[19].

22	Glutamine, L-	32.014	0.55	*Anti-inflammation*	[20].

27.	Neophytadiene	46.544	1.08	Antipyretic *Antianalgesic Anti-inflammatory* *Antimicrobial* *Antioxidant*	[21].

29.	Phytone	46.930	1.29	*Antioxidant* *Anti-inflammatory*	[22, 23].

30.	Hexadecanoate <methyl->	49.924	0.92	*Antifungal* *Anti-inflammatory*	[24, 25].

31.	Hexadecanoic acid <n->	51.394	7.21	*Antibacterial* *Antifungal* *Anti-inflammatory* *Antioxidant* Hypocholesterolemic Nematicide Pesticide Antiandrogenic Hemolytic, 5-Alpha reductase inhibitor Mosquito larvicide	[26].

33.	Palmitate <ethyl->	52.193	5.65	*Antioxidant* Anticancer	[27, 28].

35.	9,12-Octadecadienoic acid (Z,Z)-, methyl ester	55.519	0.76	Anticancer Hypocholesterolemic Nematicide Antiarthritic Hepatoprotective Antiandrogenic Hypocholesterolemic Nematicide 5-Alpha reductase inhibitor *Antihistaminic* Anticoronary Insectifuge *Antieczemic* *Antiacne*	[29, 30].

36.	Phytol	55.977	4.33	*Antimicrobial*, Anticancer *Anti-inflammatory* Antidiuretic, *Antidiabetic Immunostimulatory*	[21].

37.	Z-3,17-Octadecadien-1-ol acetate	57.593	4.32	*Antioxidant* Hepatoprotective	[31].

38.	11-(3-Ethenylcyclopentyl) undec-10-enoic acid, ethyl ester	57.814	2.22	No reported activity	

47.	Sebacic acid, bis(2-ethylhexyl) ester	74.948	0.77	*Anti-inflammatory* *Antimicrobial* *Protects skin from rash and irritation* *Restores natural skin humidity level* *Decreases hyperglycaemia*	[32–35].

^*∗*^R.T: retention time; ^*∗*^P.A (%): peak area concentration.
