# Corrigendum to “In Vitro Wound Healing Potential of Stem Extract of *Alternanthera sessilis*”

**DOI:** 10.1155/2020/2705479

**Published:** 2020-06-13

**Authors:** Katyakyini Muniandy, Sivapragasam Gothai, Woan Sean Tan, S. Suresh Kumar, Norhaizan Mohd Esa, Govindasamy Chandramohan, Khalid S. Al-Numair, Palanisamy Arulselvan

**Affiliations:** ^1^Laboratory of Vaccines and Immunotherapeutics, Institute of Bioscience, Universiti Putra Malaysia, Serdang 43400, Selangor, Malaysia; ^2^Department of Medical Microbiology and Parasitology, Faculty of Medicine and Health Sciences, Universiti Putra Malaysia, Serdang 43400, Selangor, Malaysia; ^3^Department of Nutrition and Dietetics, Faculty of Medicine and Health Sciences, Universiti Putra Malaysia, Serdang 43400, Selangor, Malaysia; ^4^Department of Community Health Sciences, College of Applied Medical Sciences, King Saud University, P.O. Box 10219, Riyadh 11433, Saudi Arabia; ^5^Muthayammal Centre for Advanced Research, Muthayammal College of Arts and Science, Rasipuram, Namakkal, Tamilnadu 637408, India

In the article titled “In Vitro Wound Healing Potential of Stem Extract of *Alternanthera sessilis*” [1], an area of overlap was identified in the control (0 h) and allantoin (0 h) panels of Figure 1(a). The authors apologize for this error, which was due to the image being saved to the incorrect file location. With the agreement of the handling editor, the revised figure is shown below.

## Figures and Tables

**Figure 1:**
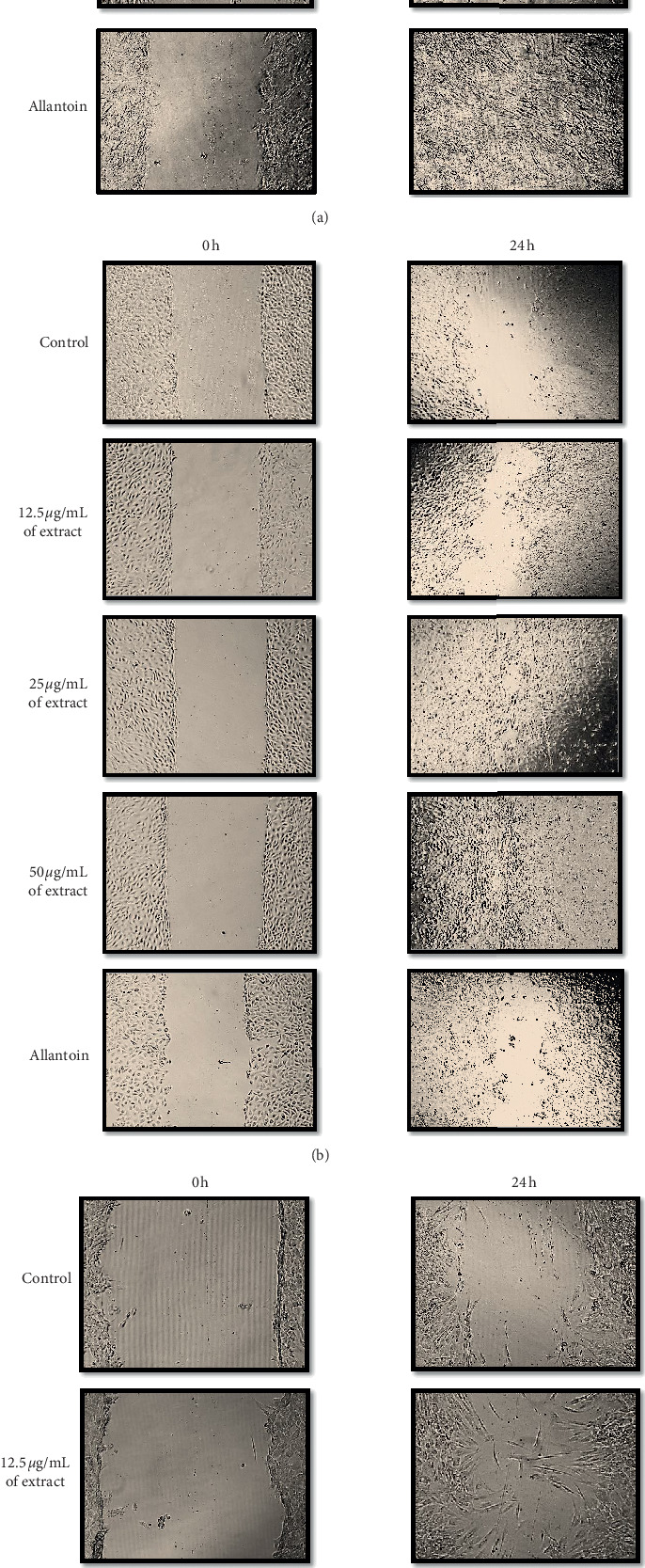
In vitro scratch assay (×40 magnification). NHDF (a), HaCaT (b), and HDF-D (c) cells were scratched and treated with and without treatment of varying concentrations of plant extract. Ethanolic extract of stem part of A. sessilis showed positive cell proliferation and cell migration as compared with control group (without treatment).
